# An investigation into the number and nature of the urgent care consultations managed and referred by community pharmacists in South-East England

**DOI:** 10.1017/S1463423620000031

**Published:** 2020-03-03

**Authors:** Linda Dodds, Barbra Katusiime, Atif Shamim, Gail Fleming, Trudy Thomas

**Affiliations:** 1 Medway School of Pharmacy, Universities of Kent and Greenwich, Chatham Maritime ME4 4TB, UK; 2 London and South East Pharmacy, Health Education England, Crawley Hospital, Crawley RH11 7DH, UK; 3 Royal Pharmaceutical Society, London E1W 1AW, UK

**Keywords:** community pharmacists, community pharmacy, urgent care

## Abstract

**Background:**

Community pharmacies are recognised as an under-utilised, accessible resource that could support the urgent care agenda. This study aimed to provide a snapshot of the number and nature of urgent care requests presented to a sample of community pharmacies in three counties in southern England, to determine how requests are managed, whether management is appropriate, as assessed by a group of experts, and whether customers receiving the care are satisfied with pharmacists’ interventions.

**Methods:**

A representative sample of pharmacists across the region was invited to keep a log-book documenting all urgent care requests over a two-week period. Data were analysed to estimate frequency and type of requests and to compare consultations in core and non-core hours. Log-book entries were scrutinised blind by an expert panel to determine appropriateness of pharmacist’s responses. Customers receiving pharmacists’ interventions were surveyed to assess satisfaction.

**Results:**

Seventeen pharmacies kept log-books detailing 432 urgent care consultations, equating to 13 consultations per pharmacy per week. Of these, 70% (*n* = 302) were dealt with by the pharmacist in-house with 30% (*n* = 130) resulting in referrals. Locum pharmacists were significantly more likely to refer to other NHS services than regular pharmacists. Over half the requests were for symptom management, skin problems presenting most commonly (38% of all symptoms presented). Forty-seven percent of consultations were considered to have ‘averted the need for other NHS services’. Pharmacists’ referral (but not assessment of urgency) was deemed appropriate by the expert panel in 90% of consultations. Ninety-five percent of customers surveyed were satisfied with the service and would use the pharmacy again.

**Conclusion:**

Extrapolating findings across the study population (approximately 4.4 million) suggests that community pharmacists manage over 11 500 urgent care consultations per week, with 8050 managed independently. These prevent approximately 5400 other NHS encounters, while also meeting customer expectations and expert panel endorsement.

## Background

Urgent care has been defined as ‘any medical or health-related condition which an individual believes they need to get help with that day’ (Centre for Pharmacy Postgraduate Education, 2016). In England, there is an increasing demand for urgent care services, putting pressure on resources in both primary and secondary care (NHS England, 2013).

People who consider they require urgent medical or health-related advice in the UK can access Accident and Emergency (A + E) departments or general practitioner (GP) services, visit a ‘walk in’ centre or ring a telephone helpline (NHS 111) for advice. NHS 111’s non-clinical call handlers use algorithms to define the nature of the problem and ascertain the most appropriate action, often referral to another service.

In mid-2016, it was estimated that NHS 111 was handling over 1.25 million calls a month, with approximately 50% of callers referred back to primary care services (Pope *et al.*, 2017). A key outcome for NHS 111 was to reduce demand on other services; however, some NHS stakeholders have suggested that it has had the opposite effect (Pope *et al.*, 2017).

The UK has around 11 600 community pharmacies (NHS Digital, 2018) situated in high street and small community locations, in supermarkets and in health centres. Many are open long hours when other health care professionals are unavailable. Many are owed by large companies but around a third are in private ownership (NHS Digital, 2018). In recent years, community pharmacists have been developing clinical services in addition to the traditional dispensing role to allow better integration and team working with the rest of the NHS. Most pharmacies now have a private consultation area specifically for confidential or sensitive discussions and are increasingly promoted as the first port of call for minor ailments and illness as well as having a key role in the promotion of a healthy lifestyle.

Because of their high accessibility, in some areas of the UK minor ailments schemes have been commissioned whereby people can receive treatment for minor conditions such as sore throat, insect bites, constipation, etc., on the NHS, saving an appointment with the person’s GP. Studies have shown that pharmacist interventions can relieve pressure on A + E and GP services, producing similar health-related outcomes for less cost (Bednall *et al.*, 2003; Baqir *et al.*, 2011; Paudyal *et al.*, 2013; Watson *et al.*, 2015). Legally pharmacists in the UK are able to make an emergency supply of a prescribed medicine when a patient has run out, provided certain criteria are met. They use their professional judgement to ensure that supply is clinically appropriate in each case. Research has shown that when free of charge, this service has high patient acceptability and potential to relieve pressure on other services and prevents patients potentially becoming non-adherent with their medication (Morecroft *et al.*, 2015; Nazar *et al.*, 2016).

Although it has been proposed that around 18% of GP consultations and 8% of A + E consultations could be managed in a pharmacy setting (Watson *et al.*, 2014), it has never been estimated to what extent community pharmacists already manage Urgent and Emergency Care (UEC) conditions, nor what types of condition the general public present to pharmacists on a daily basis. Research in 2017 showed that of the 3299 customers visiting five pharmacies, 14% requested a retail sale, with just 9% requesting advice or services, but further detail is not reported (Mackridge *et al.*, 2017).

Using a representative sample of community pharmacies across three counties in the South-East of England, this study aimed to gain understanding of the nature and number of requests that currently comprise UEC in the context of community pharmacy and to see how community pharmacists respond to these requests and how satisfied customers are with the way these requests are dealt with.

## Methods

### Setting

Community pharmacies in three counties in southern England (Kent, Surrey and Sussex, KSS).

### Study design

The study used log-books to capture the UEC requests presented to a small, representative sample of community pharmacies across the three counties of England over a two-week period to determine the nature of UEC requests. Samples of the log-book entries relating to assessment of urgency and referral were analysed by an expert panel to determine the appropriateness of referrals and estimation of urgency. A paper-based customer survey ran alongside the pharmacist UEC consultations to capture immediate customer responses to the pharmacist intervention.

### Recruitment of pharmacies

One hundred pharmacies across the south eastern counties of KSS were invited to take part with the aim of ultimately recruiting 20 pharmacies. This number was considered sufficient to provide variation in terms of location within KSS, ownership (independent pharmacy, multiple pharmacy) and hours of opening. Hours were termed core (CH), defined as Monday to Friday until 6 pm, and non-core (NCH), defined as Monday to Friday after 6 pm, Saturdays after 1 pm and all day Sundays. The number invited from each of the three counties (KSS) was proportional to the number in each county in the total sample of pharmacies in KSS (*n* = 892). Within each sample, there was further stratification to ensure that the proportion of multiples (>20 pharmacies nationwide) to independent pharmacies (20 or fewer pharmacies nationwide) was taken into consideration. Excel spreadsheet filters were used to identify eligible pharmacies in each of two main sub-groups (those providing NCH services and those only providing CH services). Simple random sampling was used to select 100 pharmacies to be invited to participate. Once a pharmacy was selected for the NCH group, it was automatically excluded from the CH group, so one pharmacy would not appear in both groups.

An invitation letter, a pharmacy information sheet and consent form were sent to all 100 representative community pharmacies by post and email. One week later, pharmacies were phoned. If interest was expressed, then a member of the research team arranged a mutually convenient time to visit to the pharmacy to obtain written consent on behalf of the pharmacy and explain the project and its instrumentation in detail. Each participating pharmacy was offered an honorarium of £500.

### Instrumentation

*Pharmacy data collection logs* were developed to record in brief all UEC presentations experienced over the study period. Each log sheet included a statement of consent and covered: pharmacist details (gender, employment status, years qualified); details of the presentation (date, time, summary of issues, including length of time issue had been present); assessment of level of urgency using a five-point scale previously devised by a focus group in another phase of this evaluation not reported here (Table 1); action taken by pharmacist was categorised as ‘advice only’, ‘advice plus sale’, ‘emergency supply’ or referral (with details), time taken to resolve; and outcome of episode of care. Each log sheet entry covered a separate episode of UEC, and the pharmacists added details of new requests consecutively. If a second pharmacist (working on the same day) was faced with a new customer request, they completed a new log sheet and added their consent and details, thus ensuring that each episode of care was attributed to a specific pharmacist.

**Table 1.** Assessment of urgency scale

Grade	Descriptor	Definition
1	Non-urgent	Long-standing problems which did not necessarily need dealing with on the same day, for example, ongoing hay fever symptoms
2	Fairly urgent	A consultation with a pharmacist ‘probably’ averted the need for other NHS services and could be appropriately managed in the pharmacy, for example, cough and cold symptoms
3	Urgent	A problem that needed resolving on the day and pharmacists’ advice ‘almost certainly’ averted a future GP visit or use of another NHS service, for example, pain after dental extraction
4	Very urgent	A problem that needed advice on the same day and pharmacists’ advice ‘definitely’ averted the use of another NHS service, for example, emergency oral contraception supply
5	Extremely urgent	A problem for which the customer should have sought other NHS services rather than a pharmacy (i.e., needed immediate referral to an emergency service) – dog bite showing signs of infection with systemic symptoms

The log sheets were piloted by three community pharmacists, and following feedback the wording used to define the levels of urgency was amended and the log sheets were compiled into a booklet for ease of use. Examples of each level of urgency were also included.

The participating community pharmacies were asked to use the log sheets to collect data on all episodes of UEC presented to the pharmacists over a two-week period in July 2016. Each episode of care was rated by the pharmacist completing it for level of urgency. CH pharmacies only collected data during core hours (mean 49 h per week) and the NCH pharmacies only during non-core hours (mean 38 h per week). Both groups collected data on Saturday mornings if they were open at that time.

A *customer survey* was designed to capture experiences of the UEC provided. Participating pharmacies were asked to recruit customers who had presented with urgent queries to complete this in the pharmacy and to ‘post’ it into a sealed box on the pharmacy counter.

The survey form and an information leaflet were devised by the research team and piloted with three non-pharmacist staff members from the Medway School of Pharmacy. As a result of piloting, changes were made to the question order. The survey asked customers to indicate using tick box responses if the UEC request was for themselves or for someone else, if they had previously used pharmacy services and if they had been referred by another professional or service to seek pharmacy advice. The survey enabled customers to rate the perceived urgency of their request and to rate their overall satisfaction with how their request was managed. They could also rate the clarity of advice given by the pharmacist. The survey explored if customers were likely to adhere to the pharmacist’s advice and if they would seek a further consultation from other NHS services. Other issues assessed were the privacy of consultations, likelihood of using pharmacy services in the future and the use of alternative urgent care providers had the pharmacy not been used.

*Expert panel*. An expert panel was convened, which consisted of a GP, nurse and pharmacist with experience of urgent care, to assess the care provided. Panel members were provided with a short data capture form to record their consensus on the level of urgency of each community pharmacy log-book entry they assessed and also whether they agreed with any referral decisions made by the pharmacist in the context of the consultation.

The expert panel reviewed a 20% sample of log-book entries. The sample was chosen by a member of administrative staff who had no connection with the study. The sample included entries from all pharmacies taking part in proportion to the number of individual entries made and covered the full range of dates and times when requests were recorded. The level of urgency of the request recorded by the pharmacist was not provided to the expert panel, who used the same scale as the pharmacists to assess the urgency of each request, blind to the pharmacist assessment. The expert panel also evaluated the appropriateness of any recorded pharmacy referrals using a three-point scale: ‘appropriate’, ‘somewhat appropriate’ and ‘not appropriate’. All assessments were carried out independently, and then consensus reached in a meeting of all three panellists.

### Data analysis

Excel spreadsheets were used for preliminary handling of datasets before all data were entered and analysed in SPSS v24 (IBM Corp. Released 2016. IBM SPSS Statistics for Windows, Version 24.0. Armonk, NY: IBM Corp.). Log sheet and customer survey data were principally quantitative but also included free-text short answer responses which were categorised with reference to the question, for example, detail of the condition presented by the customer was categorised into ‘pain’, ‘upper respiratory tract infections’, ‘skin’ and frequencies and percentages of each calculated. The data were analysed to estimate frequency and type of requests in CH versus NCH pharmacies. The outcomes of the UEC requests and the referral patterns of the participating pharmacists were analysed. Chi-square was used to assess significant differences for variables, for example, in CH and NCH. Significance was assumed when *P* < 0.05. Pharmacists’ assessments of the urgency of customer requests were compared with those of the expert panel. Agreement was determined using weighted kappa.

## Results

Twenty pharmacies were recruited to the study. However, three pharmacies agreed to participate but did not collect data due, in all cases, to unforeseen staffing issues. All three pharmacies came from the Kent region. One was a multiple pharmacy from the CH group, and two were independent pharmacies from the NCH group. For pharmacy sample characteristics, see Table 2.

**Table 2.** Characteristics of pharmacies completing log sheets compared to the pharmacies in the three counties

Characteristics	Sample *N* = 17		Pharmacies in the three counties *N* = 892	
	*n*	%	*n*	%
Kent	4	24	333	37
Surrey	6	35	218	24
Sussex	7	41	341	38
CH	10	59	607	68
NCH	7	41	285	32
Independent	8	47	398	45
Multiple	9	53	494	55
Independent CH	6	35	283	32
Independent NCH	2	12	115	13
Multiple CH	4	24	324	36
Multiple NCH	5	29	170	19

CH = pharmacies open during core hours, defined as Monday to Friday until 6 pm; NCH = pharmacies open during non-core hours, defined as Monday to Friday after 6 pm, Saturdays after 1 pm and all day Sundays.

A total of 27 community pharmacists collected data on UEC requests during the study period, of whom 7 were locum pharmacists (26%) with the remainder being the ‘regular’ pharmacist who worked in that pharmacy as their main and usual place of work.

Overall, 432 consultations were recorded (see Table 3). In the participating pharmacies, this represented a mean of 14 UEC consultations per week in CH and 10.5 per week in NCH. While the difference in the total number of consultations in multiples versus independents was not significant, there was a highly significant difference (*X* ^2^ = 82.458; df = 1, *P* < 0.001) in the number of consultations carried out during NCH in multiple pharmacies compared to the number carried out in independent pharmacies.

**Table 3.** Characteristics of pharmacies and pharmacists providing consultations during core and non-core hours

Characteristics (number)	Total consultations – core hours *N* = 285	Total consultations – non-core hours *N* = 147	Total consultations
Geography (*n* = 432) (percentage of total consultations)			
Kent (4)	122 (28)	56 (13)	178 (41)
Surrey (6)	38 (9)	40 (9)	78 (18)
Sussex (7)	125 (30)	51 (12)	176 (41)
Pharmacy type (*n* = 432) (% of total consultations)			
Multiple (9)	88 (20)	113 (26)	201 (46)
Independent (8)	197 (46)	34 (8)	231(53)
Work role of consulting pharmacist (*n* = 376) (percentage of consultations where work role stated)			
Regular pharmacist (20)	200 (53)	81 (21)	281 (74)
Locum (7)	76 (20)	19 (5)	95 (25)

*Timing of consultations*. The highest number of CH consultations/urgent requests was conducted between 10 am and 11 am, while NCH pharmacies recorded most consultations between 6 pm and 7 pm (with a similar number between 7 pm and 8 pm). The length of consultations was recorded in 414 log entries, and most consultations (48%, *n* = 200) lasted approximately 5–10 min, while 32% (*n* = 132) lasted 5 min or less. Longer times (11–20 min) were recorded for 63 (15%) consultations, and the longest consultations went on for over 20 min (*n* = 19, 16 NCH, and 3 CH).

Overall, 73% (*n* = 316) of all consultations concerned the person who made the UEC request. Seven percent (*n* = 28) were for another adult. Among consultations in which the customer’s age was recorded or estimated (*n* = 385), data showed that 17% (*n* = 65) were for children 17 and under, while over half (57%, *n* = 219) were for people aged 18 to 59, and a quarter (26%, *n* = 100) were for older persons (age 60 and over).

In terms of the type of UEC request, over half (57%, *n* = 247) were symptom-related with all types of requests presented being similar during CH and NCH (Table 4).

**Table 4.** Analysis of urgent care requests in the sample community pharmacies

Nature of consultation	CH (282) *n* (%)	NCH (146) *n* (%)	Total (432) *n* (%)
Presentation of symptoms	159 (56)	88 (60)	247 (57)
Request for prescription medicine(s)	53 (19)	28 (19)	81 (19)
Medicine-related enquiries	46 (16)	15 (10)	61 (14)
Emergency contraception	10 (4)	11 (8)	21 (5)
General health advice	9 (3)	2 (1)	11 (3)
Other	5 (2)	2 (1)	7 (2)

Further categorisation of the symptoms presented shows that they related to a wide range of clinical areas, with skin problems the most common (Table 5).

**Table 5.** Categories of urgent care symptoms presented

Symptom category	Number of symptom consultations in CH (159) (%)	Numbers of symptom consultations in NCH (88) (%)	Total (247) (%)
Skin	60 (38)	35 (40)	95 (38)
Eye	19 (12)	10 (11)	29 (12)
Musculoskeletal	16 (10)	4 (5)	20 (8)
Upper respiratory tract infections	14 (9)	5 (6)	19 (8)
Wounds	13 (8)	4 (5)	17 (7)
Gastrointestinal	11 (7)	5 (6)	16 (6)
Allergy	7 (4)	6 (7)	13 (5)
Childhood infections	4 (3)	7 (8)	11 (4)
Pain	6 (4)	5 (6)	11 (4)
Urogenital	5 (3)	4 (5)	9 (4)
Ear	4 (3)	3 (3)	7 (3)

Almost two-thirds (65%, 57 of 88) of requests were for problems of three days or shorter duration with 13% (11 of 88) of requests being made within four to seven days of the problem starting, while in five customers the problem had started over one week prior to making the UEC request. Fifteen consultations were made for long-term issues (nine for problems experienced for between one month and three months, and six for one year or more), although customers presented these as ‘urgent’ on the day.

Consultation outcome was detailed for 392 (91%) of the 432 consultations. In some cases, more than one outcome was reported for the same consultation, for example, sale of medicine to ‘tide the person over’ with a non-urgent referral. In 70% (*n* = 273/392) of the UEC presentations where an outcome was recorded, the issue was managed independently by the pharmacist with no referral made. Almost half (*n* = 201, 47%) of these consultations managed in-house were given an urgency rating of 2, 3 or 4 which by the definition given, ‘averted the need for other NHS services’.

Overall, 27% (116) of consultations were managed by provision of advice only, 42% (183) by advice plus sale of a product and 19% (81) by provision of an emergency supply of medicine, while 30% (119) received a referral.

In the consultations managed by providing advice only (116; 27%), the most common type of request was for advice on medicines (29/116; 25%) with skin conditions (21; 18%) second.

In the 183 (42%) consultations where a medicine was sold, the most common type of request was for skin (51; 28%) with eye problems second with 200 presentations (11%). In 16 consultations (4%), the pharmacist was unable to sell a product that could help with the urgent request due to its prohibitive cost to the customer.

A total of 81 (19%) of consultations involved a request for a supply of regular prescribed medication because the patient had run out. For 63 of these, the pharmacist made an emergency supply. An emergency supply was refused in 18 cases. The most common reason for not providing an emergency supply (*n* = 11/18) was when the medicine was obtained through another route, for example, if the pharmacist contacted the GP practice to obtain a prescription. There were also two requests out of the 18 refusals where an emergency supply involved a request for a controlled drug and in another case where the customer refused to pay for the emergency supply. In the remaining five cases, the reason for refusal was not recorded. There were an additional 12 emergency supplies given which were not documented as related to running out of medicines. This meant that in total 75 (17%) consultations involved an emergency supply of which 47 (63%) were made in core hours and 28 in NCH.

Independent pharmacies carried out slightly more emergency supplies (40, 53%) than multiple pharmacies (35, 47%), although this difference was not significant.

The logs indicated 119 (30%) consultations in which an outcome included a referral; of these 69 were to a GP practice, 22 to NHS 111, 10 to a walk-in centre, 9 to A + E and 9 to other services. For details of the GP referrals, see Table 6. Infection or suspected infection constituted 42 (35%) of the total number of all referrals and 42% (29) of referrals to GPs.

**Table 6.** Reason for referral to the GP practice

Reason for referral to GP	Number of referrals *n* = 69
Suspected infection	29
Medicine-related query	13
Other	9
Pain	6
Skin	5
Ran out of medicines	5
Gastric problem	2

Nine requests were referred to A + E, which included three suspected infections and two wounds.

Pharmacists working in multiple and independent pharmacies referred to GP practices similarly (29 and 33 referrals, respectively). The number of consultations managed in-house without referral was not statistically different between pharmacists working in a multiple and those working in an independent pharmacy (*P* = 0.085). Locum pharmacists were, however, statistically more likely than regular pharmacists to make a referral/not manage a UEC enquiry in-house (*P* < 0.001). However, there were no significant differences (*P* = 0.263) in the referral services used by regular or locum pharmacists.

*Urgency of requests: Customers’ ratings*. Of 207 customers who rated the urgency of their request, 58% (*n* = 121) perceived their requests as ‘fairly urgent’, 19% (*n* = 39) considered their requests to be ‘urgent’ and 19% ‘very urgent’ (*n* = 40).

*Pharmacist ratings*. Pharmacists recorded urgency in 390/432 consultations. The highest number of consultations (*n* = 133) (34%) was rated as ‘fairly urgent’ with ‘urgent’ second highest (*n* = 108, 28%). A further 14% (*n* = 56) were rated ‘very urgent’ with a further 53 (13.5%) ‘extremely urgent’. Only 40 consultations (10%) were rated as ‘not urgent’ by the pharmacists.

During CH, 37% (93) consultations were rated as ‘fairly urgent’, while during NCH, the proportion was 29% (40). During NCH, more consultations were rated ‘very urgent’ (*n* = 30, 22%) and ‘extremely urgent’ (*n* = 16, 12%) compared to during CH (*n* = 40, 16% and *n* = 13, 5%).

Of the 75 consultations where an emergency supply of medicine was made, 36% (*n* = 26) were rated as ‘very urgent’, 30% (*n* = 22) were rated as ‘urgent’, and 16% (*n* = 12) were rated as ‘extremely urgent’. An emergency supply was only made for three customer requests rated as ‘not urgent’.

Locum pharmacists were statistically (*P* < 0.001) more likely to rate consultations as urgent 61% (*n* = 43) compared to regular pharmacists (28%; *n* = 90). There was a statistically greater number (*P* < 0.001) of ‘extremely urgent’ rated consultations by multiple pharmacies (*n* = 46/186; 24%) compared to independent pharmacies (*n* = 7/204; 3%). Multiple pharmacies also rated statistically (*P* < 0.001) more consultations as ‘very urgent’ (37/186; 20%) 19) compared to independents (19/204; 9%).

*Expert panel ratings*. A total of 89 (21% of total) records of UEC events were evaluated by the expert panel. There was poor agreement between the expert panel and the pharmacists in terms of urgency ratings *K*_w_ = 0.142 (SE = 0.059; CI 0.027–0.257); however, there was strong agreement *K*_w_ = 0.955 (SE = 0.026; CI 0.904–1.006) in terms of the pharmacists’ referral (decision to refer or not) and also in terms of referral destination *K*_w_ = 0.898 (SE = 0.085; CI 0.731–1.066).

The expert panel tended to assign a lower level of urgency to emergency supply requests. Of the sample of consultations presented to the expert panel, 31 resulted in a referral. Only three of the referral decisions made by the pharmacists were considered inappropriate.

*Customer views of pharmacy advice*. Two hundred and seven customers completed the customer survey. Sixty percent (*n* = 124) were female. Almost half (49%, *n* = 102) were aged between 30 and 64 years, and 20% (*n* = 41) were 65 years or over. The remainder were below 30 years of age.

Sixty-one percent (*n* = 126) indicated that the UEC request was made for themselves, while 45 (22%) reported that the consultation was for someone else. In 35 (17%) of responses, the customer did not indicate who the consultation was for. Most respondents (75%, *n* = 154) had previous experience of using a pharmacy service. Family or friends had recommended the use of a pharmacy to 33% of respondents (*n* = 68).

Most customers (95%, *n* = 194) were satisfied with how their request was managed by the pharmacist, and the same proportion felt that the pharmacist advised them clearly about their request. Overall, 96% (*n* = 197) indicated that they would return to a pharmacy for health advice.

Seventy-six percent (*n* = 154) of survey respondents stated that they were ‘very likely’ to adhere to the pharmacist’s advice, but two reported that they were ‘not likely’ to do so. After the consultation, 43 customers (21%) believed they needed a further consultation with another healthcare professional; this follow-up consultation may have been the recommendation of the pharmacist. There were five customers who were not sure if they needed to see another healthcare professional or not and three did not respond to this question.

The privacy of consultations was rated positively by 98% of customers (*n* = 201). Over half of customers (57%, *n* = 114) indicated that they would have visited their GP and 15% (*n* = 31) contacted NHS 111 had they not received help from their pharmacy, that is, 72% of customers would have contacted another service had they not been seen by the pharmacist.

## Discussion

This study gives for the first time a detailed picture of community pharmacies’ responses to UEC consultations. If extrapolated to the region, the findings suggest that each pharmacy in KSS is undertaking, on average, 13 UEC consultations per week, with two-thirds of all consultations taking place during CH. However, because NCH pharmacies only logged UEC requests outside of CH, the CH pharmacies actually logged urgent requests over a greater number of hours in total. Seventy percent of the consultations were managed in-house by the pharmacy and at least half prevented referral to another NHS service. These findings were corroborated by the results of the customer questionnaire which showed the majority (72%) of those responding stated they would have sought other NHS services if the pharmacist had not supplied the care and advice that they did.

There are no direct comparisons with other studies looking at UEC presentations in community pharmacy generally to support these figures. The majority of studies to date have looked at commissioned minor ailments services or emergency supplies (Morecroft *et al*., 2015; Nazar *et al*., 2016). Because a considerable number of the UEC presentations here could be categorised as minor ailments, it is appropriate to make comparisons with the pharmacy minor ailment literature. Minor ailments are defined as ‘common or self-limiting or uncomplicated conditions which may be diagnosed and managed without medical (i.e., doctor) intervention’ (Watson *et al*., 2014). In such services, eligible recipients are nearly always exempt from NHS charges (Paudyal *et al*., 2013), which was not the case in this study. In 2011, *Baqir et al*. studied people who accessed a locally commissioned minor ailments scheme across in the North of England which involved 185 pharmacies. They found each pharmacy received on average 0.5 minor ailment requests per week. There are several possible reasons for the numbers in this current study being much higher. First, consultations were on any topic and not confined to minor ailments. Medication-related queries were common (14%), which may reflect an increasing appreciation by the general public that the pharmacist is an expert in medicines. The high consultation rate may also reflect a transfer in demand since 2011. Reports suggest that getting a GP appointment is becoming more difficult (NHS England 2013), while an evaluation of the impact of the NHS 111 telephone helpline noted significant increases in its use since its inception in 2014, especially during the day, with around 50% of callers being redirected to primary care (Pope *et al*., 2017).

Difference in consultation rate may also have related to the high number of emergency supplies of prescribed medicines made in this study. Emergency supplies comprised 17% of consultations, equating to 2.2 per pharmacy per week. This finding is similar to the estimation of 2.5 per week by researchers who looked specifically at emergency supplies (Morecroft *et al*., 2015).

It is of interest that there were no significant differences in the numbers or types of UEC consultations recorded in CH or NCH, indicating that similar demand exists beyond traditional pharmacy opening hours and complements existing out-of-hours health services. Morecroft *et al*. (2015) found that there were considerable numbers of requests for emergency supplies during CH. This needs to be factored into future initiatives to manage UEC activity more effectively. While there was no significant difference in the numbers of queries dealt with overall by multiple pharmacies (pharmacies with >20 branches nationally) compared with independents (<20), multiple pharmacies undertook many more consultations outside CH compared to independents. Care must be taken in interpreting this finding as the percentage of NCH multiples recruited was higher than that in the region generally.

Over half of all requests in the current study were for symptom management, with skin problems the most common. The majority of commissioned minor ailments schemes have included a high proportion of skin conditions (Paudyal *et al*., 2013) as it is a common area with which patients present (Morecroft *et al*., 2015; Tucker and Stewart, 2015). Dermatological problems were shown to be the most common ‘unnecessary’ condition presented to GPs (Hammond *et al*., 2004).

Findings from the expert panel in this study suggest that pharmacists’ referrals following consultations are appropriate. The referral destination was most often the patient’s GP, with A + E referrals occurring only rarely. There was not agreement in terms of the pharmacists’ ratings of urgency compared to the expert panel. In particular, the community pharmacists frequently rated requests for an emergency supply as being of greater urgency than the expert panel. This may reflect the pharmacists’ focus on medicines, and the importance they assign to medication adherence to manage chronic conditions. Having the person or their representative in front of them would also be likely to influence the perceived urgency by the pharmacy.

The high number of referrals related to management of infection (35% overall) is potentially a source of concern. Antibiotic resistance is a worldwide issue, and efforts are being made to stop inappropriate prescribing. Referral by the pharmacist to another provider could raise expectations about the need for an antibiotic and may make it more difficult for that provider to refuse supply. A review in 2016 by Curley *et al*. (2016) looked at community pharmacists’ referrals and showed that accuracy in identifying the presenting condition and agreement with expert medical opinion were high when the pharmacist was following a guideline or protocol, but less so without. It is possible that guidelines could be established for UEC management and referrals at national and local levels, particularly around optimal use of antibiotics. If local services wish to expand pharmacists’ roles further then schemes which train and support pharmacists to recognise, manage and refer minor verses major infection may be worth further investigation.

The study findings suggest that locum pharmacists are likely to refer more patients to other UEC services compared to the pharmacist who works regularly in that pharmacy. This highlights a potential training need for locums who may miss out on formal training sessions/opportunities provided to employed staff and is an area for focus by national and local training providers. Locums may also be less aware of local services for appropriate referral so this could be something each pharmacy could address when locums are in attendance.

High customer satisfaction with services was noted in this study with almost all saying they would use the service again. The customer sample had a similar age distribution to the customers sampled by the pharmacists. Almost half of customer completed forms and the consultations where age was stated by the pharmacists were for working age customers, indicating that this service appeared to be meeting the needs of people who may find it hard to take time out to attend appointment-based services. The widening use of community pharmacists for general health care and advice was evidenced by 17% of customers having been directed to the pharmacy by other health services and a further 33% by family or friends. Similarly, high customer satisfaction with pharmacy services has been demonstrated in earlier studies: Watson *et al*. (2015) showed resolution of symptoms following a pharmacist’s consultation ranged from 68% to 94%, while more than 90% of customers accessing a minor ailments service were willing to reuse the scheme (Paudyal *et al*., 2013).

### Limitations

The sample chosen was smaller than intended due to three pharmacies dropping out. Overall, however, there was representation from all three geographic areas, and there were no significant differences between the regions, based on the presented consultation data. While the sample was small, it was on the whole representative of the type of pharmacies across KSS although the number of pharmacies recruited in CH was slightly higher than the percentage across the region. In particular, the sample contained a greater percentage of multiples in NCH and a greater percentage in CH compared to the region generally. Even if these figures are taken to be representative of the included counties, this does not mean they reflect the picture across other parts of the UK.

The data were collected during the summer months and so may not truly reflect the nature or volume of UEC consultations throughout the year. Pressure of work may have meant that some consultations were not recorded, leading to underestimation of the volume of UEC requests presenting to pharmacies. Logs may not have been completed immediately after the presentation and this could have led to recall bias. Customers could choose whether or not to complete a survey form. This may have influenced the satisfaction ratings. Because of the anonymised nature of the customer responses, we were not able to compare customer ratings of urgency to those of the pharmacists and expert panel.

Despite these limitations, the findings give the first snapshot of what community pharmacists are dealing with in terms of UEC on a daily basis and can highlight areas where training, resources and other research may be justified in order to help community pharmacy fulfil its potential in assisting with the management of the urgent care agenda.

## Conclusion

This study evidences that community pharmacists in south-east England are effectively managing UEC requests and helping to avert visits to other NHS UEC services. They are managing conditions appropriately and to the satisfaction of their customers often with just advice and/or sale of an over the counter product.

Extrapolating the reported data across the KSS region suggests that the 892 community pharmacies in the area undertake over 11 500 UEC consultations per week, 8050 of which are managed independently, preventing approximately 5400 other NHS encounters. The data indicate that there is potential for community pharmacists to further develop their delivery of UEC services; however, this is likely to be more effective if the community pharmacists are included in local initiatives to manage UEC requests.
